# *In vitro* Cytotoxicity and Pharmacokinetic Evaluation of Pharmacological Ascorbate in Dogs

**DOI:** 10.3389/fvets.2019.00385

**Published:** 2019-11-07

**Authors:** Margaret L. Musser, Alyssa L. Mahaffey, Melissa A. Fath, Garry R. Buettner, Brett A. Wagner, Benjamin K. Schneider, Yeon-Jung Seo, Jonathan P. Mochel, Chad M. Johannes

**Affiliations:** ^1^Veterinary Clinical Sciences, Iowa State University, Ames, IA, United States; ^2^Free Radical and Radiation Biology Program, The University of Iowa, Iowa City, IA, United States; ^3^Biomedical Sciences, Iowa State University, Ames, IA, United States

**Keywords:** canine, vitamin C, high-dose, osteosarcoma, intravenous

## Abstract

**Background:** High-dose, pharmacological ascorbate (P-AscH^−^) is preferentially cytotoxic to human cancer cells *in vitro*. Investigations on the efficacy of P-AscH^−^ as an adjuvant treatment for multiple human cancers are on-going, but has yet to be formally investigated in dogs. The primary objectives of this study were to determine the pharmacokinetic (PK) profile of P-AscH^−^ in healthy Beagle dogs and the effects of P-AscH^−^ on canine osteosarcoma cells *in vitro*.

**Methods:** Eight purpose-bred, healthy, spayed female Beagle dogs, between 20 and 21 months old, and 8–10 kg were administered two doses of P-AscH^−^ (550 or 2,200 mg/kg) *via* intravenous infusion over 6 h, on separate days. Plasma ascorbate concentrations were measured at 12 time points during and after infusion for PK analysis using nonlinear mixed-effects (NLME) and non-compartmental analysis (NCA). Clonogenic assays were performed on 2 canine osteosarcoma cell lines (D-17 and OSCA-8) and canine primary dermal fibroblasts after exposure to high concentrations of ascorbate (75 pmoles/cell).

**Results:** Plasma ascorbate levels in the dogs peaked at approximately 10 mM following 2,200 mg/kg and returned to baseline 6–8 h after dosing. Minor adverse effects were seen in two dogs. Ascorbate (75 pmoles/cell) significantly decreased survival in both the osteosarcoma cell lines (D-17 63% SD 0.010, *P* = 0.005; OSCA-8 50% SD 0.086, *P* = 0.026), compared to normal fibroblasts (27% SD 0.056).

**Conclusions:** Pharmacological ascorbate is preferentially cytotoxic to canine-derived cancer cells. High levels of ascorbate can be safely administered to dogs. Further studies are needed to determine the effects of P-AscH^−^ on canine patients.

## Introduction

The potential benefit of high-dose intravenous (IV) pharmacological ascorbate (P-AscH^−^, vitamin C) for patients with cancer was first reported in the 1970s (1, 2). Subsequent clinical trials failed to show substantial improvement in outcome (3), stalling its clinical use (4). However, P-AscH^−^ continued to be a popular alternative cancer therapy (5, 6). The potential anti-cancer properties of P-AscH^−^ are being reevaluated. Several human clinical trials are examining the efficacy and safety of P-AscH^−^, for example in non-small cell lung carcinoma (NSCLC) (7), glioblastoma multiforme (GBM) (7), and pancreatic cancer (8, 9), indicating renewed interest in the therapeutic use of P-AscH^−^ for human cancer patients.

Ascorbic acid is a weak acid with p*K*_a1_ of about 4.2 (8). Thus, at the near-neutral pH of mammalian biology, greater than 99.9% will be present as the monoanion, i.e., ascorbate. The biochemistry for the functions of vitamin C is principally the biochemistry of ascorbate. In this work, we use the term pharmacolocial ascorbate, P-AscH^−^, to distinquish the functioning of ascorbate as a drug from its biochemistry as vitamin C (10, 11). When at normal, healthy physiologic levels ascorbate functions as a reducing agent and donor antioxidant (12); however, at supraphysiological concentrations, P-AscH^−^ can produce high fluxes of H_2_O_2_ *via* its oxidation (8, 9, 13). The cytotoxic mechanism of P-AscH^−^ appears to be due to these fluxes of H_2_O_2_ and, in part, to alterations in cancer cell oxidative metabolism, allowing for increased steady-state levels of ${\text{O}}_{2}^{•-}$ and H_2_O_2_ within tumor cells (7, 14, 15). These alterations disrupt intracellular iron metabolism, sensitizing cells to the effects of the oxidation of P-AscH^−^ (7, 14, 15). Multiple *in vitro* studies have confirmed the cytotoxic effects of P-AscH^−^ on human and murine tumor cells, and the sparing effect on normal cells (10, 11, 14, 15).

Human studies of P-AscH^−^ have determined that therapeutic, cytotoxic plasma levels (≥≈20 mM) cannot be achieved through oral administration (PO) (16). Therefore, IV or intraperitoneal (IP) injection is required (16, 17). In addition, high-dose IV P-AscH^−^ infusions (15–125 g), given over hours, are required to achieve and maintain proposed therapeutic plasma levels due to the rapid clearance of ascorbate (7, 16–19). The optimal dose of P-AscH^−^, frequency, and duration of administration have yet to be determined (20).

Pharmacological ascorbate is generally well-tolerated and appears to have no dose-limiting toxicities in humans (6). Adverse effects that have been described include vomiting, diarrhea, weight loss, leukopenia, and neutropenia (6, 7, 10, 21).

Canine pharmacokinetic (PK) analysis of PO ascorbate, but not P-AscH^−^, has been scientifically evaluated (22). In 4 Greyhounds that were treated with 1 g of ascorbate PO or IV, peak ascorbate plasma concentrations were significantly greater when ascorbate was administered IV (mean 0.33 ± 0.06 mM) compared to PO (mean 0.03 ± 0.01 mM) and was achieved within 6 min, falling rapidly back to baseline (mean 0.01 mM) within 6 h (23). Recently, seven dogs were reported to have received various doses of IV ascorbate for the adjuvant treatment of multiple cancers with minimal side effects (24). Data regarding the PK of P-AscH^−^ in dogs, and if P-AscH^−^ would be a feasible treatment option for our canine patients, are lacking.

The purpose of this study was two-fold: (1) determine the PK profile of P-AscH^−^ in healthy Beagle dogs; and (2) determine the effects of P-AscH^−^ on canine osteosarcoma cells. Given the lack of significant adverse side effects reported in humans, we hypothesized that IV P-AscH^−^ would be safe for use in dogs and that *in vitro*, P-AscH^−^ would cause significant cytotoxicity to canine osteosarcoma cells. The PK data presented here can guide the development of treatment protocols that are feasible for use in the veterinary clinical setting.

## Methods

### Experimental Animals

Eight purpose-bred, spayed female Beagles, between 20 and 21 months old, and 8–10 kg were used in this study. The dogs were obtained from Ridglan Farms, Mt. Horeb, WI. The use of the Beagles for this study was approved by the Iowa State University Institutional Animal Care and Use Committee. Compliance with the US National Research Council's Guide for the Care and Use of Laboratory Animals was maintained throughout the study. The Beagle dogs were group-housed in the same conditions and fed the same Royal Canin® diet for the duration of the study. Prior to infusions of P-AscH^−^ and collection of blood samples, dogs were fasted overnight with free choice water available at all times.

### Ascorbate Administration

Two doses of injectable L-ascorbic acid, 500 mg/mL (Mylan Institutional LLC, Canonsburg, PA), were administered on two separate days. (Although labeled as Ascorbic Acid for Injection, the contents are at near-neutral pH, thus vials contain ascorbate.) A low dose (550 mg/kg) was administered on the first trial day. This dose was escalated to 2,200 mg/kg for administration on the second trial day, as previously reported (24). At least a two-day washout period was given between each dose. Administration of ascorbate was performed through a 20 gauge, peripheral short IV catheter in the left or right cephalic vein of each dog. Pharmacological ascorbate was combined with sterile water to achieve a 500 mg ascorbate per mL solution with a targeted osmolarity between 500 and 900 mOsm/L (7). The infusion was given over 6 h (12.5 mL/h for the 550 mg/kg dose; 50 mL/h for the 2,200 mg/kg dose) *via* an IV infusion pump in order to achieve a steady-state plasma ascorbate concentration. Dogs were continuously monitored during the infusion and intermittently for 24 h post infusion for adverse clinical signs.

### Plasma Ascorbate Sample Preparation and Analysis

To evaluate plasma ascorbate concentrations throughout and after infusion, blood was sampled at 12 time points: immediately prior to the ascorbate infusion, a baseline venous sample was obtained; venous blood was sampled at 0.5, 1, 3, 5, and 6 h during the infusion period; and at 6.5, 7, 8, 10, 12, and 16 h during the post-infusion period. Prior to the higher dose infusion (2,200 mg/kg), and at the 6 h mark of the higher dose infusion, arterial samples for blood gas analysis were also obtained. All venous blood collection was performed *via* a Cavafix® (B Braun Medical Inc., Breinigsville, PA) sampling catheter placed in either the left or right lateral saphenous vein, to the level of the caudal vena cava, following manufacturer recommendations (25). At each time point, 2 mL whole blood samples were drawn into a Vacutainer® PST™ Lithium Heparin (Becton Dickinson, Franklin Lakes, NJ) green top tube and promptly stored on ice until centrifugation. Blood was centrifuged for 20 min at 4°C and 2,000 rpm (671 ***g***). Plasma was collected and stored at −80°C until analysis (26). Arterial samples for blood gas analysis were obtained *via* an AirLife^TM^ Reduced Heparin Arterial Blood Sampler (CareFusion).

Plasma ascorbate quantification was conducted at The University of Iowa using two different assay systems as previously described (27, 28). Briefly, a plate reader (SpectraMax® M3, Molecular Devices, Sunnyvale, CA) was used to measure baseline and physiological levels of ascorbate present in the plasma samples (28). To quantify the high pharmacological levels of ascorbate a second assay using an Implen Nanophotometer (Implen GmbH, Munich, Germany) microvolume UV-Vis spectrometer was used. With little required sample processing, this assay, developed at The University of Iowa (27), allows rapid assessment of ascorbate concentrations in plasma ranging from 2.9 to 35 mM.

Arterial blood gas samples were processed by the Iowa State University Clinical Pathology Lab using a RAPIDPoint® 500 Blood Gas Analyzer (Siemens Medical Solutions USA, Inc., Malvern, PA). Additional complete blood counts, chemistries, and urinalyses were performed in conjunction with another study taking place during the same time period.

### Pharmacokinetic Modeling

Time-courses for the concentration of ascorbate in blood plasma following low-dose and high-dose IV infusion were analyzed simultaneously using the stochastic approximation expectation maximization (SAEM) algorithm implemented in the Monolix Suite 2018R2 (Lixoft, France). Data below the lower limit of quantification (LLOQ) were modeled by adding to the likelihood function a term describing the probability that the true observation lies between zero and the LLOQ. Individual model parameters were obtained *post-hoc* using the mean of the full posterior distribution, as previously described for non-linear mixed-effects (NLME) models in animal health (29). Convergence of the SAEM algorithm was evaluated by inspection of the stability of the fixed and random effect parameter search and the log-likelihood estimate after the exploratory period of the algorithm (i.e., after 1,000 iterations of SAEM). Standard goodness-of-fit diagnostics, including individual predictions *vs*. observations, the distributions of weighted residuals, and normalized prediction distribution errors were used to assess the performances of the candidate models (30–32). Prediction distributions from 1,000 Monte Carlo simulations were used to evaluate the ability of the final model to reproduce the variability in the observed pharmacokinetic data. Residual error estimates from the mathematical models were used as supportive information for evaluation of lack of fit. Normality and independence of residuals were assessed using histograms, quantile-quantile plots, and autocorrelation of conditional weighted residuals. For converging models with satisfactory goodness-of-fit diagnostics, model selection was based on the Bayesian information criteria (BIC) and the precision of the model parameter estimates.

For completeness, non-compartmental analysis (NCA) of ascorbate PK was performed using PKanalix (Monolix Suite 2019R1, Lixoft, France). Standard PK parameters were generated for individual dogs, as follows:

- Area under concentration-time curve, AUC_INF_;
  - A linear trapezoidal linear rule was used to estimate the area under the ascorbate time-curves
- Maximum concentration, C_MAX_;
- Steady state concentration, C_SS_;
- Steady state volume, V_SS_;
- Clearance, Cl;
- Time of maximum concentration, T_MAX_;
- Half-life, T_1/2_;
- Elimination rate constant, λ_z_;
  - Computed by linear regression of the logarithmic concentration vs. time curve during the elimination phase

### *In vitro* Ascorbate Analysis—Cell Lines and Reagents

Two canine osteosarcoma cell lines were obtained, D-17 (American Type Culture Collection, Rockville, MD, USA) and OSCA-8 (Kerafast, Inc., Boston, MA), and grown in pyruvate free DMEM 10% fetal bovine serum (FBS). Canine primary dermal fibroblasts were obtained as a normal tissue control (Cell Biologics, Chicago, IL). Cell lines were grown in proprietor complete fibroblast media (M2267 for dermal fibroblasts) containing 1 mM pyruvate. Cells were kept in either 21 or 4% O_2_ at 37°C, 5% CO_2_, in a humidified atmosphere.

For cell culture studies, ascorbate stock solutions, 1 M in water, with the pH adjusted to 7.0 with NaOH were prepared from L-ascorbic acid (Macron Fine Chemicals, Avantor Performance Materials, Inc., Center Valley, PA) and stored in the dark at 5°C in sealed glass test tubes until use (11). The ascorbate stock concentration was verified spectrophotometrically. Appropriate volumes of the stock to achieve 50 pmole/cell (5 mM) and 75 pmol/cell (7.5 mM) were added to culture media for 1 h as previously described (7, 11).

### *In vitro* Ascorbate Analysis—Clonogenic Survival Assay

To determine the effect of P-AscH^−^ on the reproductive integrity, D-17 and OSCA-8 canine osteosarcoma cells were plated at 80,000 cells per 60-mm dish. Seventy-two hours after plating, one dish was counted to determine the number of cells on the plate. The volume of media in the dishes was adjusted so that cells were treated with a range of stock ascorbate, 2.5 pmoles/cell (0.25 mM)−50 pmoles/cell (5 mM). Ascorbate was dosed per cell as previous literature has demonstrated that H_2_O_2_ and ascorbate toxicity depends on cell number/density (15, 33, 34). After 1 h of P-AscH^−^ treatment both floating and attached cells from control and treated dishes were collected using 0.25% trypsin (GIBCO). Trypsin was inactivated with media containing any floating cells, centrifuged at 1,200 rpm (335 ***g***) for 5 min and resuspended in fresh media. Cell counts were made with a Coulter Counter (Beckman Coulter Z1) and the cells were plated at various densities and allowed to grow for 10–12 days in complete media at 21% O_**2**_. Subsequently, the cells were stained with Coomassie Blue dye, and colonies of >50 cells on each plate were counted and recorded. Surviving fraction was determined as the number of colonies per plate divided by the number of cells initially added to that plate. Normalized surviving fraction was determined as the surviving fraction of any given clonogenic plate from a treatment group divided by the average surviving fraction of the control (untreated) plates within a given experiment.

The clonogenic experiment comparing osteosarcoma cells to canine fibroblast cells were performed similarly with the following exceptions. Optimal conditions for normal fibroblasts is 4% O_2_ (normal physiologic condition) and proprietary media (complete fibroblast media M2267) which contains sodium pyruvate, known to scavenge H_2_O_2_ (15). In addition, normal canine fibroblasts will only form clones when plated on lethally irradiated feeder cells (see below). Therefore, in experiments comparing the reproductive viability of P-AscH^−^ on cancer cells *vs*. fibroblasts, the dose of P-AscH^−^ was increased to 75 pmol/cell (7.5 mM) and all cultures, including the sarcoma lines, were treated in identical fibroblast media at 4% O_2_ and plated onto feeder cells.

### *In vitro* Ascorbate Analysis—B1 Feeder Layer Cells

Chinese hamster fibroblasts designated as B1 (passages 20–40) were maintained at 21% oxygen in high glucose DMEM 1× media containing L-glutamine and sodium pyruvate and supplemented with 10% FBS, 20 mmol/L HEPES, pH 7.3, 1× MEM non-essential amino acids. The feeder layer was prepared 24 h prior to any clonogenic survival assay by irradiating the cells with 30 Gy of X-rays at a dose rate of 27 Gy/min then plating in the cloning dishes at 100,000 cells per dish. During clonogenic survival assays comparing P-AscH^−^ treatment of cancer cell lines to normal canine fibroblasts, all cloning plates were grown using a feeder layer and each clonogenic experiment had two extra dishes plated with feeder cells alone to ensure no colony growth at the end of the 10- to 12-day incubation.

### Statistical Analysis

The clonogenic survival analysis was done using a minimum of three cloning dishes per experimental condition, and the experiments were repeated a minimum of three times on separate occasions. Statistical analyses were done using the GraphPad Prism software. Statistical significance was determined using one-way ANOVA and Dunnett's *multiple comparisons* test was performed. Error bars represent ± standard error of the mean, and statistical significance was defined as *P* < 0.05.

The Wilcoxon signed rank test was used to examine the difference between the pre- and post-ascorbate administration for each of nine safety parameters evaluated by blood gas. A *P*-value < 0.05 was considered significant. Statistical analysis was performed using R (version 3.5.2) (R Foundation for Statistical Computing, Vienna, Austria).

## Results

### Safety

The ascorbate infusion was well-tolerated by all dogs, with one dog experiencing a grade I veterinary cooperative oncology group-common terminology criteria for adverse events (VCOG-CTCAE) vomiting during the higher dose infusion, and another showing VCOG-CTCAE grade I nausea/ptyalism (35). No changes on complete blood count or chemistry panels were noted during the course of the study for any dog. Blood gas analysis revealed statistically significant changes in pre- and post-ascorbate calcium, sodium, potassium, chloride, bicarbonate, partial pressure of carbon dioxide, anion gap, lactate, and glucose measurements (Table 1). The pH was not significantly different pre- and post-ascorbate administration.

**Table 1:** Arterial blood gas and electrolyte abnormalities observed in eight Beagle dogs after receiving pharmacological ascorbate (2,200 mg/kg).

**Parameter**	**Median pre-ascorbate**	**Median post-ascorbate^**a**^**	**Median difference (post - pre)**	**IQR difference^b^**	***P*-value^c^**	**Canine normal^d^**	**Ascorbate and sterile water blank**
Ca (mmol/L)	1.37	1.40	0.030	0.030	0.034	1.23–1.40	Decreased out of range
Na (mmol/L)	146	152	6.00	4.25	0.007	143–151	224x
K (mmol/L)	3.56	3.39	-0.170	0.170	0.015	3.60–4.70	<1
Cl (mmol/L)	114	107	-7.00	1.50	0.013	109–117	N/A
HCO_3_ (mmol/L)	21.3	22.7	1.40	0.900	0.015	19.0–25.0	N/A
pCO_2_ (mmHg)	37.1	39.9	2.80	3.15	0.023	29.0–45.0	N/A
Anion gap (mmol/L)	15.6	28.4	12.8	4.10	0.007	12.0–21.0	N/A
Lactate (mmol/L)	0.64	8.64	8.00	2.10	0.007	0.43–2.10	Increased out of range
Glucose (mg/dL)	107	51.5	-55.5	23.0	0.014	62.0–115	246
pH	7.37	7.37	0.00	0.040	1.00	7.34–7.45	N/A

a *Blood was drawn at the 6-h mark immediately following cessation of pharmacological ascorbate infusion*.

b *IQR: interquartile range*.

c *P-values for differences were obtained from the Wilcoxon signed-rank test (significance level = 0.05)*.

d *Canine Normal displays a generally accepted normal range for healthy dogs for each parameter for the RAPIDPoint 500 analyzer (36, 37)*.

### *In vivo* Pharmacokinetics

**Figure 1:**
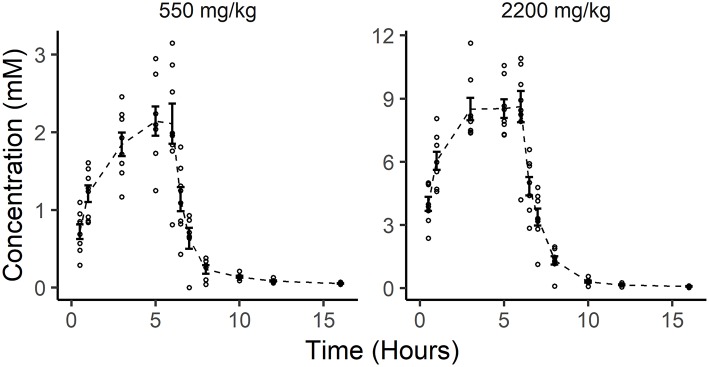
*In vivo* pharmacokinetics of two doses of pharmacological ascorbate in eight healthy Beagle dogs. Distribution of individual ascorbate concentrations (small open circles) are plotted alongside the mean ascorbate concentrations (dashed line) and standard error (bars) for all observations at each time point following ascorbate infusions (550 and 2,200 mg/kg).

The mean initial plasma ascorbate concentration for the eight dogs at the 550 mg/kg P-AscH^−^ dose (≈4 g) was 0.02 mM (± 0.01 mM). Over the course of the 6-h infusion, this peaked to 2.14 mM (± 0.54 mM). Once the infusion was completed, ascorbate levels fell sharply below therapeutic levels, and returned to near baseline by 6 h post infusion (Figure 1). The mean initial plasma ascorbate concentration for the eight dogs at the 2,200 mg/kg dose of P-AscH^−^ (≈15–21 g) was 0.02 mM (± 0.01 mM). Over the course of the 6-h infusion, this peaked to 8.6 ± 2.1 mM. Once the infusion was completed, ascorbate levels fell sharply below proposed therapeutic levels, and returned to near baseline by 6 h post infusion (Figure 1).

**Figure 2:**
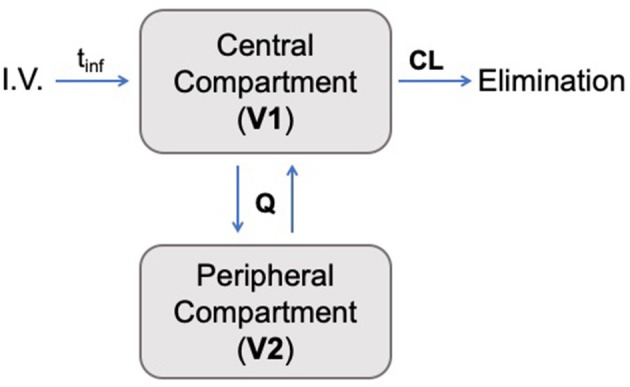
Schematic pharmacokinetic representation of the two-compartment model developed in this study. Ascorbate was administered intravenously (**I.V**.) with an infusion time denoted by **t**_**inf**_. The transfer between the two compartments is governed by intercompartmental transfer parameter **Q**, and the drug's elimination was determined by the clearance from the central compartment (**CL**).

**Figure 3:**
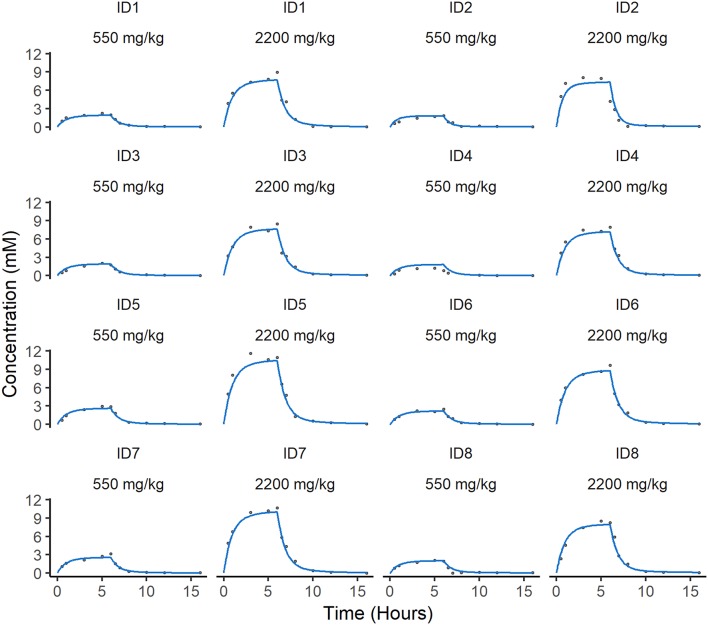
Individual predicted concentration of ascorbate in plasma. Individual predicted curves (blue) are plotted against the individual measurements of ascorbate (gray circles) over time. There are two individual fits for each animal dependent on dose. The difference between the individual predictions and observations defines the residual error in the model, which is low in these cases.

A two-compartment mammillary disposition model with first-order elimination and zero-order absorption following IV infusion best described the PK of ascorbate in plasma (Figure 2). A combined error model with an additive and a proportional error term was used to account for the residual noise in ascorbate measurement. The robustness and predictive performances of the final model were supported by the inspection of the standard goodness-of-fit diagnostics (Supplemental Figure 1). Overall, the model was able to reproduce the individual experimental data for the two dosing schedules with excellent accuracy, as shown by the quality of the individual fits (Figure 3).

Using standard mathematical equations for a two-compartment PK model (38), the elimination half-life of ascorbate in canine plasma can be estimated at ~3.5 h. Population PK parameter estimates and their related variances are summarized in Table 2. For all model parameters, the precision of the final estimates was considered satisfactory (RSE ≤ 25%). The systemic total body clearance (CL) of ascorbate was estimated to be low to moderate (2.0 L/h, or 0.24 L/kg/h), according to previous definition (39), while the steady-state volume of distribution was estimated to be relatively small (2.69 L or 0.32 L/kg), with the central compartment occupying most of the distribution of ascorbate in dogs. The global extraction ratio of ascorbate (E) calculated as CL/Q [with cardiac output Q (mL/kg/min)] approximated by the formula: Q = 180 × BW^−0.19^(39), was estimated to be low (E = 0.03).

**Table 2:** Model parameter pharmacokinetic estimates as determined by the stochastic approximation expectation-maximization algorithm.

**Parameter**	**Symbol**	**Unit**	**Point estimate**	**RSE (%)**	**IIV (%)**
Clearance	CL	L/h	2.04	3.18	6.86
Central Volume	V1	L	1.96	5.99	11.9
Inter-compartmental Clearance	Q	L/h	0.16	15.0	–
Peripheral Volume	V2	L	0.76	24.7	46.6

*RSE, Relative Standard Error of the Estimate; IIV, Inter-individual Variability*.

**Figure 4:**
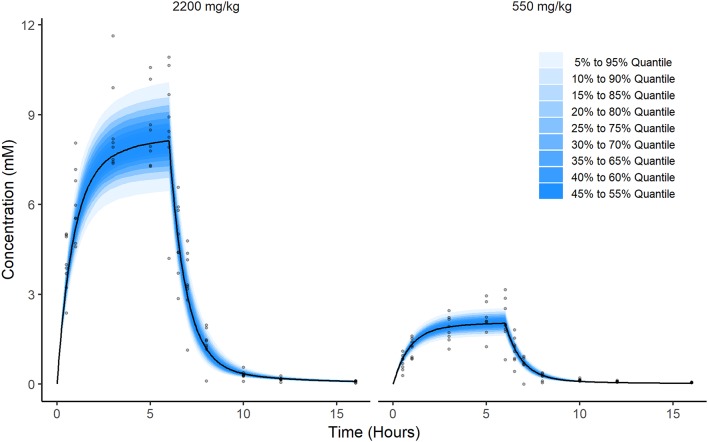
Prediction of ascorbate distribution. These figures were produced by Monte Carlo simulation from the model fit. Briefly, a population of 2,000 individuals were simulated. Half were virtually administered a dose equal to 2,200 mg/kg and the other half were administered a dose equal to 550 mg/kg. Then, at each time point from 0 to 16 h in steps of 0.05 h, i.e., (0, 0.05, 0.1, 0.15, …,15.95, 16), the quantiles of the resulting simulations were calculated and plotted along with observations for comparison (gray dots).

Prediction distributions (from the 2.5th to 97.5th percentile) derived from 1,000 Monte Carlo simulations confirmed the good performances of the final selected model which was able to reproduce the variability in the observed disposition kinetic data of circulating ascorbate following IV infusion dosing (Figure 4).

The NCA PK parameters are collated in Table 3. The mean maximum ascorbate plasma concentration was estimated as 9.23 ± 1.33 mM for beagles administered 2,200 mg/kg P-AscH^−^ and 2.26 ± 0.61 mM for beagles administered 550 mg/kg P-AscH^−^. Peak plasma concentrations were observed around a median value of 5.5 ± 1.0 h. Mean clearance and steady state volume estimates were unremarkable (2.05 ± 0.35 L/h and 3.52 ± 1.54 L respectively). The median elimination half-life was estimated at 3.73 ± 1.47 h. All NCA PK parameters agree well with their NLME counterparts.

**Table 3:** Non-compartmental analysis pharmacokinetic parameters.

	**Mean PK parameters** **±** **SD**						**Median PK parameters** **±** **SD**	
Group	AUC_INF_	C_MAX_	C_SS_	V_SS_	Cl	λ_z_	T_MAX_	T_1/2_
Unit	mM*h	mM	mM	L	L/h	1/h	h	h
All	33.7 ± 22.4	5.74 ± 3.73	5.37 ± 3.74	3.52 ± 1.54	2.05 ± 0.35	0.22 ± 0.12	5.5 ± 1.0	3.73 ± 1.47
2,200 mg/kg	54.5 ± 8.8	9.23 ± 1.33	9.01 ± 1.47	2.60 ± 0.51	1.96 ± 0.16	0.25 ± 0.13	6.00 ± 1.36	3.87 ± 1.44
550 mg/kg	12.8 ± 3.1	2.26 ± 0.61	2.06 ± 0.51	4.44 ± 1.52	2.15 ± 0.46	0.18 ± 0.08	5.00 ± 0.52	3.73 ± 1.49

*SD, standard deviation; AUC_INF_, median area under curve; C_MAX_, maximum concentration; C_SS_, steady state concentration; V_SS_, steady state volume; Cl, clearance; T_MAX_, time of maximum concentration; T_1/2_, half-life; λ_z_, elimination rate constant*.

### Clonogenic Assay

**Figure 5:**
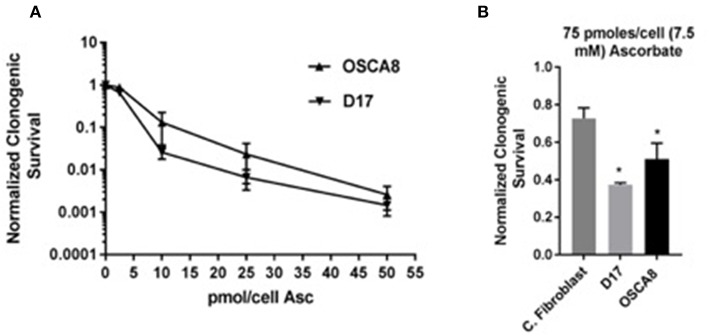
Pharmacological ascorbate treatment results in selective clonogenic cell death in canine osteosarcoma cell lines. **(A)** Significant dose dependent decreases in clonogenic survival for two osteosarcoma cell lines (D-17 and OSCA-8) following treatment with 0.25–50 pmol/cell ascorbate at 21% O_2_ DMEM no pyruvate 10% FBS media. **(B)** Significant survival differences between two osteosarcoma cell lines (D-17 and OSCA-8) *vs*. primary canine fibroblasts after treatment with 75 pmol/cell ascorbate in fibroblast media 4% O_2_. *Denotes statistical significance.

To test the hypothesis that treatment with P-AscH^−^ decreases clonogenic survival in canine osteosarcoma cancer cells, D-17 and OSCA-8 were treated with 0.25–50 pmol/cell ascorbate for 1 h. In both cell lines, P-AscH^−^ treatment resulted in significant (*P* < 0.001), dose-dependent decreases in clonogenic cell survival compared to untreated controls (Figure 5A). To test the hypothesis that P-AscH^−^ causes differential susceptibility in osteosarcoma cells *vs*. normal canine dermal fibroblast, again clonogenic survival assay was performed. Optimal growing conditions for dermal fibroblast include proprietary media containing 1 mmol pyruvate and 4% O_2_. Both O_2_ and pyruvate have been demonstrated to affect the production and removal of H_2_O_2_ with ascorbate treatment (15), therefore clonogenic assay was done using 75 pmol/cell P-AscH^−^ for 1 h and optimal fibroblast growing conditions for both the cancer cell lines and normal fibroblast cells. Treatment with P-AscH^−^ demonstrated significant differential susceptibility in osteosarcoma cells *vs*. normal fibroblast by decreasing clonogenic survival in both osteosarcoma cell lines (D-17 63%, SD = 0.010, *P* = 0.005; OSCA-8 50%, SD = 0.086, *P* = 0.026), more than normal fibroblasts (27%, SD = 0.056) (Figure 5B).

## Discussion

Based on the plasma ascorbate concentration analysis reported herein, it has been determined that it is safe to administer P-AscH^−^ (2,200 mg/kg) to healthy dogs. Using an infusion rate of 50 mL/h of a solution containing 500 mg ascorbate per mL, *in vivo* peak plasma ascorbate concentrations reached up to 10 mM and were found to be dose-dependent. To our knowledge, this is the first comprehensive characterization of ascorbate PK in dogs. A two-compartmental mammillary disposition model with first-order elimination and zero-order absorption after IV infusion dosing best described the available PK data. Parameter estimates of the final NLME model suggest that ascorbate has a rather low extraction ratio (0.03) but a small volume of distribution, resulting in a short elimination half-life in dogs, in agreement with previous findings from the human literature (19).

Adverse effects that were previously noted in human studies, including vomiting, diarrhea, and weight loss, were minimal in our canine subjects (21). During the infusion of both doses, and for at least 24 h post infusion, only one dog vomited. All dogs remained bright, alert, and responsive, had a good appetite following the infusion, and maintained their body condition. This suggests that P-AscH^−^ will be well-tolerated in healthy dogs, even at elevated doses.

It is worth noting that in the present study, mild changes on blood gas analysis were noted after administration of 2,200 mg/kg P-AscH^−^ (Table 1). There are several possible explanations for these findings, the first of which is analytical interference. A significant elevation in anion gap and hyperlactatemia were observed 6 h into the infusion of the higher dose P-AscH^−^ (2,200 mg/kg) in all eight dogs. Ascorbic acid has been shown to interfere with blood gas electrodes that use oxidase for amperometric methods of detection (namely lactate and glucose) (40, 41), including the Siemens analyzer used in this study (42). Additionally, when a comparable solution of ascorbate and sterile water was run alone, elevations in lactate were detected (Table 1), suggesting the apparent hyperlactatemia is most likely due to an electrode analytical interference and may not be clinically relevant.

Alternatively, hyperlactatemia is a reported side effect of multiple drugs, the mechanism of which is varied but does include interference with the Cori cycle, inhibition of mitochondrial protein synthesis, inhibition of the electron transport chain (ETC), and uncoupling of oxidative phosphorylation (43). Recent research with P-AscH^−^ in humans revealed that several of the mitochondrial ETC complexes may be inhibited during treatment (7), possibly contributing to the hyperlactatemia. The true cause of the hyperlactaemia in the Beagles is unknown, and may be a combination of analytical interference and more insidious causes. Awareness of this possible elevation is extremely important as use of ascorbate becomes prevalent in clinically ill dogs and humans. Future studies are needed to determine if hyperlactatemia is truly due to analytical interference or if it might be more clinically relevant.

Elevated bicarbonate and hypernatremia were noted and expected, as the formulation of P-AscH^−^ is buffered by sodium and bicarbonate. Expected compensatory hypochloremia and high pCO_2_ were also observed. The elevation of sodium and decreased chloride may also be attributed to analytical inference, as erroneous hypernatremia and hypochloremia have been noted previously following the use of P-AscH^−^ (40). Glucose, although expected to be high (44), was found to be decreased in all 8 Beagles. This was attributed to the fast prior to and during the infusion of P-AscH^−^.

Hypokalemia was likely due to increased diffusion of ascorbate into cells, followed by secondary increase in Na/K-ATPase activity and rapid intracellular potassium uptake (dogs synthesize ascorbate from glucose via the glucuronic pathway and absorb it *via* passive diffusion, while in people ascorbate requires facilitated diffusion or active transport) (45, 46). This is contrary to an expected hyperkalemia that has been noted in humans and has been attributed to an analytical interference (40). This discrepancy may be attributed to the different mechanisms by which ascorbate is taken into cells in dogs compared to humans.

Calcium at the upper end of the reference interval post-treatment was an unexpected finding, as in people ascorbate acts as a weak calcium chelator, possibly leading to hypocalcemia (47). However, ascorbate has been shown to interfere with calcium analysis, much like lactate, usually resulting in false elevations (40), which may have occurred in our study. In addition, high doses of oral ascorbate have previously been found to cause hypercalcemia in broiler chickens and hens, likely due to altered calcium metabolism (either enhancement of intestinal absorption or resorption of bone) (48), which may be a phenomenon unique to species that can synthesize ascorbate, such as dogs.

Most of the blood gas analysis changes, although mathematically significant, were considered to be mild and are likely due to analytical interference caused by high concentrations of ascorbate in the plasma. Knowledge of these possible alterations, however, are important so that overzealous correction of these temporary, and likely clinically insignificant, changes does not occur to the detriment of the patient. Further analysis is necessary to make conclusions about the causes of these observed changes.

The two assays for the determination of plasma ascorbate concentrations used in this study have not previously been reported in a canine trial setting. The plate reader assay has been validated with canine, porcine and human plasma (28). The 0 h results (≈20 μM) from this study fall within the established detection range for the assay and correspond with values that were found in the validation study (28). To date, the Implen Nanophotometer has not been used to quantify plasma ascorbate in canines (49). A recent study confirmed the ability to use UV spectroscopy to rapidly quantify high concentrations (up to 35 mM) of human plasma ascorbate, with minimal processing (27). Using this same assay on our canine plasma samples detected ascorbate concentrations that corresponded with those found in human and murine studies (7, 17, 21). The novel use of these quantification methods in this study provides proof of concept for future studies.

*In vitro*, following treatment with P-AscH^−^ ranging from 0.25 to 50 pmole/cell, the clonogenic cell survival of both OSA cell lines (D-17 and OSCA-8) was significantly decreased compared to untreated controls in a dose-dependent manner (Figure 5A). Similarly, the clonogenic survival of both cell lines after treatment with 75 pmole/cell (7.5 mM) of P-AscH^−^ was significantly decreased compared to normal canine fibroblasts (Figure 5B). This is similar to human OSA *in vitro* studies, which showed a dose-dependent decrease in cell growth following exposure to P-AscH^−^ (range of doses up to 1 mM) and a sparing of normal control cells; the most significant growth effects were found at the 1 mM concentration (50, 51). Similar findings have been confirmed for a variety of human tumors (7). Recently, the *in vitro* effects of P-AscH^−^ on canine melanoma cell lines have also been investigated, and show similar, dose-dependent findings with IC_50_ values (those that reduce the survival of each cell line by 50%) between 3.6 and 9.9 mM (52).

Utilizing human cell lines, ascorbate concentrations of 1–7 mM are required for cytotoxicity and additional cell kill is achieved with higher doses (10). However, the suggested target level of plasma ascorbate that is believed to be most effective for humans is ≈20 mM (7). When the Beagles received a dose of 2,200 mg/kg over 6 h, plasma levels peaked at approximately 10 mM around the 6 h mark. Although this is above the *in vitro* cytotoxic threshold of 7.5 mM, it is below the target dose in humans. However, it is not known if the human target level of 20 mM is required, or if lower plasma concentrations may be equally successful. In addition, *in vitro* data cannot be directly correlated with an expected cytotoxic dose *in vivo*, as conditions in these two situations are different (15). Specifically, for *in vitro* P-AscH^−^ treatment, the cell cultures contain 1 mM of pyruvate which is known to remove cytotoxic H_2_O_2_, potentially interfering with the main mechanism of action of P-AscH^−^, and decreasing the observed effects of P-AscH^−^ *in vitro* (53).

The level of intracellular labile iron also influences the cytotoxicity of P-AscH^−^ (7, 14). In general, normal cells have a lower labile iron pool compared to cancerous cells, potentially explaining the sparing effects of P-AscH^−^ on normal cells. The level of labile iron in the OSA cells is unknown, but it is possible that they have a relatively decreased amount of iron, impacting the cytotoxicity of P-AscH^−^ *in vitro*. Similarly, this would impact cytotoxic levels *in vivo* should a particular patient have a tumor with inherently less labile iron, be anemic (54), have endogenous or exogenous exposure to glucocorticoids (55), or have circadian drops in iron (56), complicating administration and assessment of an expected cytotoxic dose. *In vivo* tumor analysis of P-AscH^−^ levels are required to fully assess the concentrations of P-AscH^−^ achievable and if these levels are clinically effective.

*In vitro* analysis also fails to replicate the *in vivo* microenvironment that exerts a significant influence on the achievable intracellular levels of therapeutics. Thus, direct correlations between the cytotoxic *in vitro* dose of P-AscH^−^ and that which will be cytotoxic *in vivo* is not possible. The *in vitro* information can guide *in vivo* experiments and the interpretation of results. Our *in vitro* data suggest that P-AscH^−^ is cytotoxic to canine OSA cell lines, providing proof of concept for additional investigations in tumor-bearing dogs.

Interestingly, it has been determined that pancreatic cancer, NSCLC, and GBM cells treated with ascorbate are more sensitive to concurrent or subsequent treatment with ionizing radiation and chemotherapy, while normal cells were spared (7, 57). In a murine model, it has been shown that pharmacologic ascorbate enhances pancreatic tumor cell radiation cytotoxicity while offering potential protection from radiation damage to normal surrounding tissue (57). This indicates that adjuvant ascorbate combined with other cancer therapies, may be more advantageous than ascorbate alone, or that synergism between treatments may imply a lower dose of P-AscH^−^ would still be effective (58). Further research into this area is needed to determine the best use of ascorbate in our canine patients.

The most advantageous dosing strategy for P-AscH^−^ is also unknown (20). Recent literature report that the clearance of P-AscH^−^ in humans following a 60-g dose was 6.0 L/h (19). In light of this fast elimination from the body, a bolus loading dose followed by a maintenance infusion of P-AscH^−^ was recommended to maintain P-AscH^−^ concentrations in the potential cytotoxic range (>20 mM) (19), but this dosing was not studied further. In the current Beagle study, a clearance of 2.0 L/h was identified. This slower clearance may indicate that an *in vivo* cytotoxic dose is achievable in dogs.

This study evaluated the use of P-AscH^−^ in healthy Beagle dogs. It is acknowledged that oncology patients may have significantly different plasma dispositions of P-AscH^−^ due to their disease that may influence dosing, PK parameters, toxicity, and efficacy of this treatment in the clinical setting. Clinical patients will be of a variety of breeds, who may process P-AscH^−^ differently from Beagles due to genetic differences. In addition, clinical patients will be on a variety of medications, including chemotherapy, which may cause unintended drug-drug interactions, or alter the PK parameters, toxicity, and efficacy of P-AscH^−^. Additional studies in tumor-bearing dogs are needed to fully evaluate the PK parameters and impact of P-AscH^−^ in canine oncology patients.

In summary, the findings presented here give a biological basis for the efficacy of ascorbate *in vitro* and an understanding of the pharmacokinetics of P-AscH^−^ in healthy dogs. Together, they provide compelling evidence to investigate the concurrent use of ascorbate with standard of care chemotherapy and radiation therapy in tumor-bearing dogs. The combined *in vitro* and *in vivo* results suggest that P-AscH^−^ may be a safe, reasonable adjuvant therapeutic option for dogs diagnosed with cancer, specifically osteosarcoma, and that cytotoxic doses of ascorbate may be achievable *in vivo*. Further studies are necessary to determine the optimal dose to achieve cytotoxic levels in tumor tissue, ideal dosing strategies, and to confirm the safety profile when combined with traditional oncologic therapies.

## Data Availability Statement

The datasets generated for this study are available on request to the corresponding author.

## Ethics Statement

The animal study was reviewed and approved by the Iowa State University Institutional Animal Care and Use Committee. Compliance with the US National Research Council's Guide for the Care and Use of Laboratory Animals was maintained throughout the study.

## Author Contributions

MM, AM, MF, GB, BW, JM, and CJ were involved with experimental design, data analysis, and manuscript writing. MM and AM obtained all *in vivo* experimental data. GB and BW analyzed all blood samples for vitamin C levels. MF designed, executed, and analyzed the data of the *in vitro* cell culture experiments. BS, Y-JS, and JM interpreted the pharmacological data and prepared the PK model. BS and Y-JS assisted with manuscript writing and peer review.

### Conflict of Interest

The authors declare that the research was conducted in the absence of any commercial or financial relationships that could be construed as a potential conflict of interest.
