# Supplementary material for: In vitro Cytotoxicity and Pharmacokinetic Evaluation of Pharmacological Ascorbate in Dogs
Source: Front Vet Sci. 2019 Nov 7;6:385. doi: 10.3389/fvets.2019.00385 (PMC6854015; doi:10.3389/fvets.2019.00385)
Supplement: Supplementary file 1 [file Table_1.DOCX]

Supplementary Material


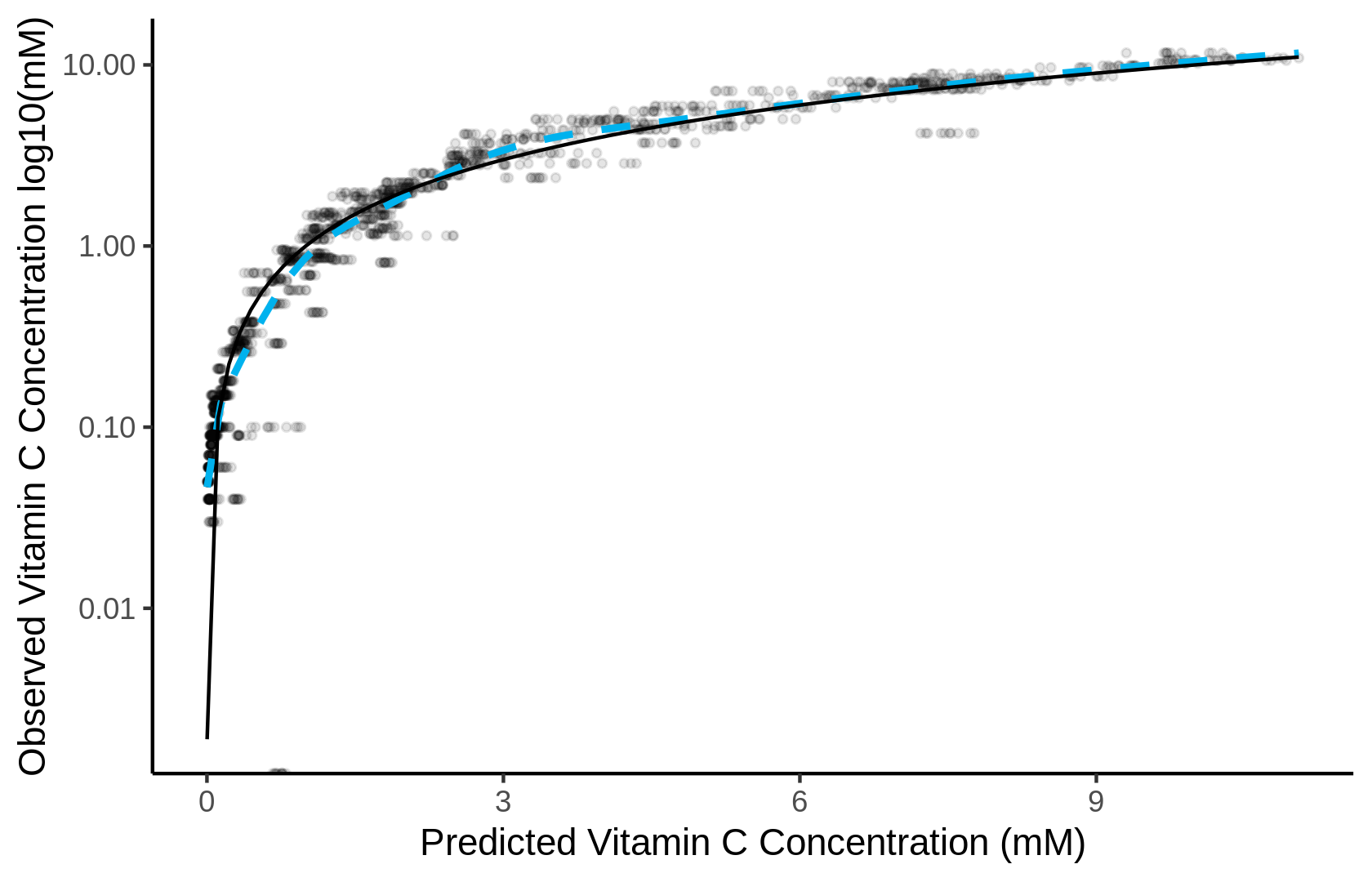


Supplemental Figure 1. Assessment of quality of model fit. Individual observations and individual predictions are plotted against each other to analyze the quality of model fit. Points have semi-transparent grey fill to reduce visual distortions due to over-plotting. The dashed cyan line is the spline best-fit for the data and the solid black line is the line of perfect fit (i.e. observation exactly equals prediction). The difference between the dashed and solid line indicates the degree to which the model is misspecified. Here, the misspecification is very low. Predictions are displayed in log10 scale to allow analysis of model fit across the full range of observations (min = .03, max = 11.64).
